# Acute pulmonary embolism following corticosteroid administration in acute severe ulcerative colitis with gastrointestinal bleeding: A case report

**DOI:** 10.3389/fcvm.2022.1018462

**Published:** 2022-10-11

**Authors:** Xuhan Liu, Xia Peng, Chunyan Rong, Weihua Zhang

**Affiliations:** ^1^Department of Cardiovascular Medicine, The First Hospital of Jilin University, Changchun, China; ^2^FAW General Hospital, Changchun, China

**Keywords:** ulcerative colitis, infliximab, pulmonary embolism, corticosteroids, VTE

## Abstract

**Background:**

Ulcerative colitis often leads to gastrointestinal bleeding and venous thromboembolism (VTE). At present, there is no clear conclusion about anticoagulant therapy for these patients. Treatment for ulcerative colitis usually includes 5-aminosalicylic acid, corticosteroid, and biologics. Acute ulcerative colitis, usually caused by infection, is usually severe and can be life-threatening.

**Case presentation:**

We report the development of VTE in a patient with severe acute ulcerative colitis who experienced gastrointestinal bleeding following hormonal therapy. The patient's indicators suggested that his blood was hypercoagulable and that his prognosis was poor.

**Conclusion:**

Patients with ulcerative colitis are prone to form VTE. The use of corticosteroids during treatment increases the risk of thrombus. Anticoagulation strategy for patients with ulcerative colitis after gastrointestinal bleeding is a problem that needs to be discussed.

## Background

An ulcerative colitis is a chronic illness that affects the colon and rectum (1). It usually causes bleeding in the colon, diarrhea, and tenesmus, as well as low abdominal pain (1). In the last few decades, ulcerative colitis incidence has been rising worldwide (2). The majority of patients at risk for such aggressive disease behavior are young and non-smokers (3). On colonoscopy, they show deep ulcerations and elevated inflammatory biomarkers (3). The risk of VTE increases in patients with inflammatory bowel disease (IBD) (3). Patients with IBD are more likely to suffer from VTE events, which carry significant morbidity and mortality (3). Here we present a case of a patient with acute pulmonary embolism following corticosteroid administration in acute severe ulcerative colitis with gastrointestinal bleeding.

## Case presentation

A 41-year-old Chinese man was diagnosed in January 2018 with ulcerative colitis. Colonoscopy revealed diffuse hyperemia and edema of the sigmoid colon and rectum mucosa about 30 cm from the anal margin, with multiple erosions and ulcers of different sizes. The bottom is covered with white moss. Peripheral mucosa hyperemia and edema. The rectum shows a patchy pattern of fused ulcers. Thereafter, the patient took mesalazine 1.5 g irregularly once a day. He still intermittently appeared pus blood stool. The patient did not smoke or drink in the past, and had no history of allergy, other diseases or trauma. There was no family history of thrombosis.

**Figure 1:**
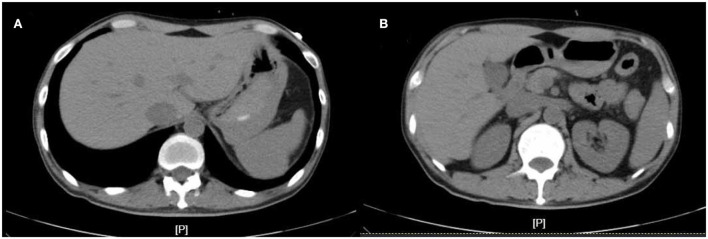
The abdominal computed tomography (CT) at admission. **(A,B)** are CT images at different levels.

**Figure 2:**
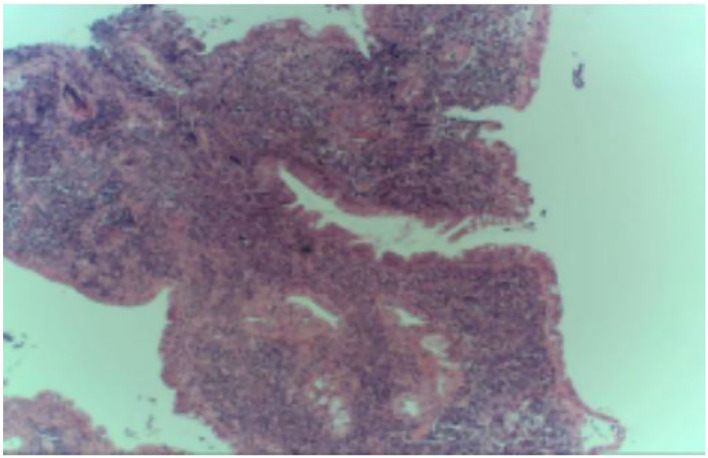
Immunohistochemistry of pathological tissues.

In April 2022, he was admitted to the First Hospital of Jilin University for 3 months of intermittent lower abdominal pain, 1 month of aggravation accompanied by black stools, and 1 day of blood in stool. Prior to this, he had a fever at night for 20 days with the highest temperature of 40°C. He took methylprednisolone 40 mg orally once a day. But he still had a fever. He underwent an emergency colonoscopy. The abdominal computed tomography (CT) showed high-density shadow in the stomach and multiple lymph nodes in the abdominal cavity (Figure 1). The examination results indicated diffuse hyperemia and edema in descending colon, sigmoid colon and rectum. Diffuse erosion and ulceration were seen, some of which were large sheets and longitudinal, and mucosal exfoliation. Extensive spontaneous oozing of the mucosa is seen. Large amounts of fresh blood, blood clots and fecal matter were seen in the lumen. Endoscopic biopsy revealed chronic inflammation of the mucosa, accompanied by inflammatory activities, local ulceration, and a large number of acute and chronic inflammatory cells infiltrated in the interstitium. Cytomegalo virus DNA (CMV) (+), Epstein-Barr virus encoded RNA (EBER) (-) (Figure 2). And his diagnosis was clear: ulcerative colitis, Mayo 3 (severe). Due to the patient's presence of deep ulcerations and extensive disease, adequate hormone therapy was given in the context of adequate anti-infective therapy. Methylprednisolone 60 mg once a day intravenously. In combination, Infliximab 400 mg was administered intravenously on the third day of admission, combined with methylprednisolone 40 mg iv. After the treatment of acid inhibition, fluid infusion, anti-virus, liver protection, fluid infusion, albumin supplement and blood transfusion, the patient improved (Table 1). During the period of active gastrointestinal bleeding, the patient had severe hemoglobin reduction and hemodynamic instability. In order to prevent the complications of aggravating anemia, multiple blood transfusions were required for treatment. After the treatment of blood transfusion, hemostasis and anti-inflammatory, the gastrointestinal bleeding improved and the hemodynamics became stable gradually. The patient was not taking antiplatelet and anticoagulant therapy during the bleeding event. After discharge, the hormone was regularly reduced and taken orally.

**Table 1:** The changes in significant laboratory test results at the first admission.

**Parameter**	**Values Day 1**	**References value**	**Unit**
WBC	8.04	3.50–9.50	10^9/*L*^
MO%	0.14	0.03–0.10	
EO%	0.001	0.004–0.08	
HGB	58	130–175	g/L
PLT	442	125–350	10^9/*L*^
FBG	5.73	1.8–4.0	g/L
ALT	652.1	9.0–50.0	U/L
Γ-GT	207.4	10.0–60.0	U/L
ALP	246.6	45.0–125.0	U/L
Cholinesterase	1,936	4,629–11,500	U/L
ALB	27.8	40.0–55.0	g/L
Novel coronavirus nucleic acid test	–	–	
D dimer	1.47	0.00–0.50	mg/L FEU
TSH	0.102	0.35–4.94	ulU/ml
FT3	1.84	2.43–6.01	pmol/L
CRP	86.57	0–3.5	mg/L
Epstein-barr virus nucleic acid quantification	4.98E3		copies/ml
EB virus core antigen IgG antibody	1.936	< 1.090	s/co
EB virus capsid antigen IgG antibody	5.760	< 1.090	s/co
ESR	47	0–15	mm/1 h
PPD experiment	negative	negative	

WBC, white blood cell; MO, monocytes; EO, eosinophils; HGB, hemoglobin; PLT, platelet; FBG, fibrinogen; ALT, Alanine aminotransferase; Γ-GT, γ glutamyl transpeptidase; ALP, Alkaline phosphatase; ALB, albumin; TSH, thyroid stimulating hormone; FT3, free T3; FT4, Free T4; CRP, C-reactive protein; ESR, erythrocyte sedimentation rate; PPD, tuberculin pure protein derivative.

**Figure 3:**
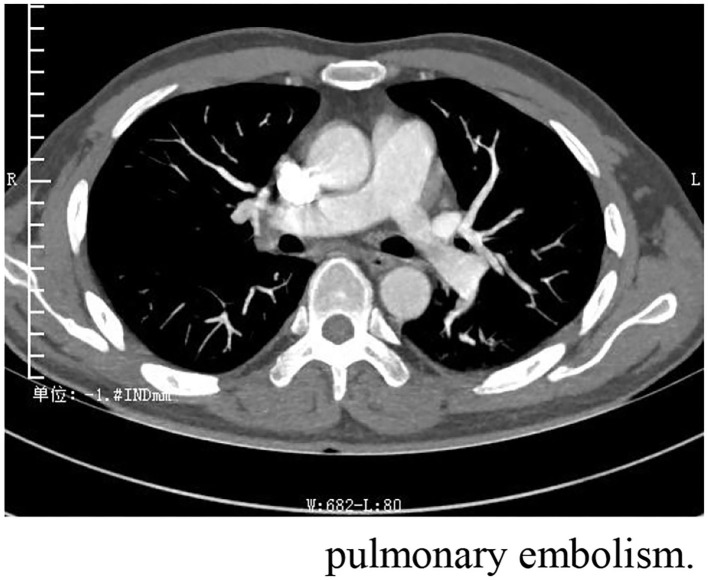
Pulmonary artery CTA suggests strip filling defects were observed in the right upper, middle and lower lungs and in the branches of the left upper and lower arteries, which is bilateral pulmonary embolism.

One month after discharge, he was readmitted with dyspnea for 7 days. At this time, he took methylprednisolone 4 mg orally once a day. Physical examination: body temperature 36.5°C, respiration 20 beats/min, pulse 105 beats/min, blood pressure 124/64 mmHg, cyanosis and sweating. Edema of both lower extremities. Pulmonary artery CTA (Computed Tomography Angiography) suggests bilateral pulmonary embolism. Ultrasonography of both lower limb veins showed thrombus in the lower segment of left popliteal vein, posterior tibial vein and fibular vein (acute stage). Left calf intermuscular vein thrombosis (acute stage). Since the patient's symptoms of dyspnea were relatively severe and pulmonary artery CTA results (Figure 3) were inconsistent with the symptoms, pulmonary ventilation and pulmonary perfusion were recommended. The probable cause of the low thrombotic burden and disproportionate respiratory failure in this patient was a small thrombus with multiple pulmonary artery branches that could not be seen directly in pulmonary CTA, resulting in more severe respiratory failure. However, due to the epidemic, it is not possible to conduct tests. This patient was diagnosed with pulmonary embolism (PESI score low risk group). The patient's condition improved after anticoagulation and oxygen therapy (Table 2).

**Table 2:** Laboratory test on the readmission.

**Parameter**	**Values Day 1**	**References value**	**Unit**
WBC	10.11	3.50–9.50	10^9/*L*^
EO%	0.008	0.004–0.08	
HGB	93	130–175	g/L
PLT	321	125–350	10^9/*L*^
D dimer	2,190	100–600	ng/ml
Cholinesterase	3,991	4,629–11,500	U/L
ALB	29.6	40.0–55.0	g/L
Novel coronavirus nucleic acid test	–	–	
CRP	4.18	0–3.5	mg/L
ESR	75	0–15	mm/1 h
Plasma antithrombin III	69.5%	80.0–130.0	%
Anti cardiolipin IgM antibody	147	0–12	MPL
Determination of anti-β 2-glycoprotein I antibody (IgG/A/M)	48	0–20	RU/ml

## Discussion and conclusion

Ulcerative colitis is a common chronic inflammatory disease of the colon and rectum, resulting from an immune reaction that is dysregulated against intraluminal antigens when the host is genetically predisposed. Peak incidences occur between 15 and 45 years of age. Approximately 20% of patients experience a severe flare during this disease, requiring inpatient care (1). Acute severe ulcerative colitis (ASUC) is diagnosed using the modified Truelove and Witts criteria, combining a bloody stool frequency ≥6 per day with at least one sign of systemic toxicity, such as a heart rate of >90 bpm, a temperature of >37.8°C, a hemoglobin level of <105 g/l and/or an erythrocyte sedimentation rate (ESR) (4). Several other risk factors for salvage therapy include a high number of bowel movements, a high serum C-reactive protein (CRP) level and a low serum albumin level (5). The patient was diagnosed with severe acute ulcerative colitis and his indicators suggested a poor prognosis.

Superimposed infections (an infectious colitis that develops over the existing ulcerative colitis) are a well- known cause of acute severe flare-ups of ulcerative colitis (6). Besides the classic enteropathogens such as Campylobacter and Escherichia coli, infections with Clostridium difficile and cytomegalovirus (CMV) should always be ruled out as a cause in cases of severe steroid refractory colitis (6). The quantitative results of cytomegalovirus and tuberculosis in this patient were negative, and other etiological tests were also negative. However, colonoscopic pathology revealed a positive DNA result for cytomegalovirus. The positive etiological test of EPstein-Barr (EB) virus does not exclude the possibility of EB virus inducing acute severe ulcerative colitis. Therefore, the patient was treated with antiviral therapy. At the same time, the patient was not excluded from the possibility of bacterial infection, so antibacterial treatment was also carried out.

It is thought that corticosteroids reduce the activation of NFκB and downregulatory cytokines such as TNF (7). Infliximab (IFX) is an anti-TNF chimeric monoclonal antibody injected into a vein. Patients with acute severe ulcerative colitis (ASUC) who fail to respond to adequate intravenous hormone therapy for 3–5 days should consider switching to IFX therapy. IFX is the most effective salvage treatment. With persistently elevated CRP levels >50 mg/l (>476 nmol/l), infliximab seemed to be required at high dosages. As a strategy, it could be recommended giving 5 mg/kg of infliximab and monitoring serum. The patient was first treated with glucocorticoid. After a long period of hormone therapy, the patient's symptoms did not improve, so he was replaced with IFX treatment. After treatment with IFX, his CRP and ESR decreased, and his symptoms were relieved. Therefore, the treatment was considered effective.

Deep vein thrombosis (DVT) and pulmonary embolism (PE) are both parts of VTE, which has a high risk of death and morbidity. The general mortality rate for lower limb DVT is 6%, and the rate for pulmonary embolism is 20% (8). Inflammatory bowel disease (IBD) is an independent risk factor for the development and recurrence of venous thromboembolism (VTE) (9, 10). VTE in patients with IBD is a multifactorial event that can be influenced by both hereditary and acquired factors, which can coexist, thereby multiplying the individual prothrombotic risk (3, 11). The acquired risk factors include active disease, oral contraceptive use, hyperhomocysteinaemia, dehydration, prolonged immobilization, infections, indwelling catheters, obesity and long-distance travel (12). VTE is most frequently caused by mutations of the prothrombin gene, G20210A, factor V Leiden mutation, and methylenetetrahydrofolate reductase, C677T (12). VTE in IBD is multifactorial and poorly understood, which is associated with hypercoagulability not because of one mechanism, but rather because of complex interactions between multiple systems (10). These include higher levels of inflammatory cytokines, acute phase reactants, pro-coagulants, and low levels of anticoagulants (13). Studies indicate that coagulation factors V, VII, VIII, X, XI, XII, fibrinogen and thrombin formation are all elevated (14). Furthermore, IBD patients have increased platelet counts (thrombocytosis) in active disease states (15). Research has suggested that IBD patients have lower protein S and antithrombin levels during active disease states, which are crucial drivers (16). The patient had increased fibrinogen and platelets, and decreased antithrombin III, which was consistent with the changes related to IBD hypercoagulable state. He had a DVT in the left leg, a bilateral pulmonary embolism, indicating that he had developed VTE. Given the morbidity and mortality associated with venous thromboembolism (VTE), international guidelines recommend routine subcutaneous low-molecular-weight heparin (LMWH) for hospitalized (17). However, the patient was hospitalized for the first time due to gastrointestinal bleeding. If anticoagulation treatment is given, gastrointestinal bleeding will be induced again, and even life-threatening. The patient was not found to have VTE at that time, so anticoagulant therapy was not given. However, the patient was readmitted with VTE and was given effective anticoagulant therapy on the second admission. This led us to consider whether anticoagulant therapy should be given to patients cured of gastrointestinal bleeding after discharge. After taking rivaroxaban tablets orally for 2 months, the patient was considered to have chronic recurrent ulcerative colitis because of the symptoms of abdominal pain and pus and blood stool. So he stopped taking it. How to avoid the risk of bleeding if anticoagulant therapy is given? The risks/benefits of prophylactic anticoagulation need to be evaluated at the time of treatment. And there are no trials that have been tested in people with IBD. Patients with such ulcerative colitis and pulmonary embolism should be given a full course of anticoagulant therapy as soon as possible after the gastrointestinal bleeding has been clearly hemostatic (e.g., no mucous, pus or blood stool, no drop in hemoglobin). IBD patients should always consider hydration, vitamin deficiencies that reduce homocysteine levels, graded pressure socks, or pneumatic devices, as well as early postoperative activity (18).

A common treatment for mild UC, mesalamine inhibits platelet activation spontaneously and by thrombin (19). However, the association with reduced risk of VTE has not been well-established (19). However, corticosteroids often have a number of adverse side effects, including an increased risk of VTE. The mechanism is linked to elevated cortisol levels, as a result of increased production of procoagulation factors and impaired fibrinolytic capacity (20). On the other hand, immunosuppressive medications like anti-TNFα biologics are believed to reduce the risk of VTE.

Anticoagulant therapy is an effective treatment for PTE in preventing thrombosis recurrence and promoting the body's own fibrinolytic mechanism to dissolve the formed thrombus. Upon diagnosis of acute PTE, anticoagulant therapy should be initiated. During this patient's anticoagulant therapy, he received enoxaparin sodium. According to the 2021 guideline (21), the fixed anticoagulant subcutaneous dose of VTE should be 250 IU/kg every 12 h. Our patient weight ~ 75 kg, so it should be 18,750 IU/12 h. Due to the patient's previous history of gastrointestinal bleeding and low hemoglobin, we reduced the dose to 4,000 U /12 h subcutaneous injection. Therefore, the patient could not determine the specific cause of thrombosis. What is clear, however, is that the patient's VTE was not caused by prolonged immobilization. Because the patient himself has clarified that he has regular exercise every day. Considering that the blood hypercoagulable state caused by UC may lead to thrombosis, we did not conduct further coagulation study for the patient to exclude hereditary causes.

It is expected that the incidence of ulcerative colitis is still on the rise, as well as the number of patients diagnosed with ASUC. UC causes the hypercoagulable state of blood through multiple systems, thus forming VTE. Corticosteroids causes increased production of procoagulant factors and impaired fibrinolysis. How to prevent UC from becoming ASUC and anticoagulant therapy strategies for UC are issues that need further discussion.

## Data availability statement

The raw data supporting the conclusions of this article will be made available by the authors, without undue reservation.

## Ethics statement

Written informed consent was obtained from the participant for the publication of this case report. Written informed consent was obtained from the individual(s) for the publication of any potentially identifiable images or data included in this article.

## Author contributions

XL: methodology, investigation, formal analysis, and writing-original draft. XP: conceptualization, methodology, and visualization. CR: investigation, formal analysis, and writing-review and editing. WZ: project administration and supervision. All authors contributed to the article and approved the submitted version.

## Conflict of interest

The authors declare that the research was conducted in the absence of any commercial or financial relationships that could be construed as a potential conflict of interest.

## Publisher's note

All claims expressed in this article are solely those of the authors and do not necessarily represent those of their affiliated organizations, or those of the publisher, the editors and the reviewers. Any product that may be evaluated in this article, or claim that may be made by its manufacturer, is not guaranteed or endorsed by the publisher.
